# Pain Processing and Vegetative Dysfunction in Fibromyalgia: A Study by Sympathetic Skin Response and Laser Evoked Potentials

**DOI:** 10.1155/2017/9747148

**Published:** 2017-09-28

**Authors:** Marina de Tommaso, Katia Ricci, Giuseppe Libro, Eleonora Vecchio, Marianna Delussi, Anna Montemurno, Giuseppe Lopalco, Florenzo Iannone

**Affiliations:** ^1^Neurophysiopathology of Pain Laboratory, Basic Medical Science, Neuroscience and Sensory System Department, Bari Aldo Moro University, Bari, Italy; ^2^Department of Emergency and Organ Transplantation, Bari Aldo Moro University, Bari, Italy

## Abstract

**Background:**

A dysfunction of pain processing at central and peripheral levels was reported in fibromyalgia (FM). We aimed to correlate laser evoked potentials (LEPs), Sympathetic Skin Response (SSR), and clinical features in FM patients.

**Methods:**

Fifty FM patients and 30 age-matched controls underwent LEPs and SSR by the right hand and foot. The clinical evaluation included FM disability (FIQ) and severity scores (WPI), anxiety (SAS) and depression (SDS) scales, and questionnaires for neuropathic pain (DN4).

**Results:**

The LEP P2 latency and amplitude and the SSR latency were increased in FM group. This latter feature was more evident in anxious patients. The LEPs habituation was reduced in FM patients and correlated to pain severity scores. In a significant number of patients (32%) with higher DN4 and FIQ scores, SSR or LEP responses were absent.

**Conclusions:**

LEPs and SSR might contribute to clarifying the peripheral and central nervous system involvement in FM patients.

## 1. Introduction

According to new diagnostic criteria, fibromyalgia (FM) is a chronic and heterogeneous disorder characterized by diffused pain, tenderness on palpation, fatigue, nonrestorative sleep, and cognitive dysfunction [1, 2]. Although the pathophysiology of FM is not well known, an abnormal pain modulation, generating increased central sensitization phenomena, is considered as peculiar [3]. FM patients generally present with a normal neurological examination, but a small subgroup of these patients shows some abnormalities, like slight distal sensory deficits. In the last three decades, laser evoked potentials (LEPs) have been demonstrated to be a useful tool for selective evaluation of nociceptive pathways in experimental pain models as well as in central and peripheral neurological diseases [4], particularly in neuropathic pain [5]. In fact, these potentials can be recorded from the vertex (“late component”) and temporal (“early component”) cerebral zones by selective activation of A*δ* and C mechanic-thermal nociceptors in the superficial layers of the skin [6]. The studies employing LEPs in FM patients confirmed increased responses from the cortical areas devoted to nociceptive stimuli processing [7–9], such as in other chronic pain syndromes, like migraine [9]; FM subjects also showed reduced habituation under repetitive painful stimulation, which seems linked to enhanced phenomena of central sensitization [10]. On the other hand, studies based on skin biopsy and nociceptive responses elicited by concentric electrode [11] or CO_2_ laser stimulation [12] demonstrate small fiber dysfunction as showed by reduction of epidermal fiber density and nociceptive responses amplitude in FM patients [13, 14]. Such studies may suggest a possible role of small fiber neuropathy in fibromyalgia pain. Phenotypic heterogeneity seems to emerge among FM groups, which would include patients with different involvement of peripheral and central nervous system dysfunction [12]. In addition, FM diagnosis may be attributed to patients with positive history but inactive rheumatic and immune diseases, which could be the cause of small fibers damage [15]. Other methods exploring small vegetative fibers function showed abnormalities in FM patients. The sympathetic skin response (SSR) is a slow wave, generated in deep layers of the skin, resulting from activation of the sudomotor sympathetic efferent fibers. In good methodological conditions, SSR appears a simple, effective means of assessing sympathetic sudomotor outflow in central and peripheral system disorders [16]. The SSR recorded from both palms and soles of FM patients showed significantly longer latency than those of healthy subjects [17], while the amplitude of SSR has been reported reduced in some studies [18] or normal in others [17]. Recently, Ozkan et al., employing artificial neural networks, demonstrated that SSR could be a new auxiliary diagnostic method to use in the FM diagnosis [19]. The utility of SSR in the diagnosis of small fibers neuropathy is limited [20, 21], so the latency abnormalities described in FM patients might not be attributed to a peripheral sensory nerves dysfunction, and its basis needs to be fully explained.

In the present study, the aims were (1) to correlate LEPs and SSR parameters, including habituation, in a cohort of FM patients and (2) to correlate results from both LEPs and SSR with FM clinical features.

## 2. Methods

### 2.1. Subjects

In our case-control study we enrolled 50 consecutive patients with fibromyalgia syndrome (46 females; median age: 47.37 ± 13.15 years). Patients with FM were recruited from the Neurophysiopathology of Pain Unit of the Bari Policlinico General Hospital from January 2015 to January 2016. FM was diagnosed by history-taking and clinical assessment according to the 2010 American College of Rheumatology criteria for FM [2]. The exclusion criteria were scholar age of less than 8 years and any peripheral or central nervous system (CNS) diseases, including spinal cord diseases and radiculopathies, psychiatric disease, diabetes, active and/or positive history for thyroid insufficiency, renal failure, autoimmune diseases, inflammatory arthritis, systemic connective tissue disease, present or previous history of cancer, as well as use of drugs acting on the CNS or chronic opioid therapy. Patients taking analgesics were instructed to avoid analgesic use 24 h prior to the laser evoked potentials examination in order to avoid any effect on LEPs amplitudes [22]. Psychiatric disorders were defined according to the criteria of the Diagnostic and Statistical Manual of Mental Disorders, 4th ed (DSM-IV).

The ICHD-III criteria [23] were applied to identify and exclude patients with migraine, a very frequent comorbid disorder in FM patients [24] since this is characterized by neurophysiological abnormalities that may overlap with fibromyalgia frame [12, 25]. Patients with chronic tension-type headache were also excluded, although a history of episodic tension-type headache was not an exclusion criterion. Patients selected for the study were assigned to CNS-acting drug treatments only after both LEPs and clinical assessment were carried out. Thirty age- and sex-matched healthy volunteers, selected among the Hospital and University staff, without any neurological, medical, and psychiatric problems, were recruited as control subjects. The study was approved by the Policlinico of Bari Ethics Committee and conducted in accordance with the Declaration of Helsinki. Written informed consent was obtained from all study participants.

### 2.2. Clinical Examination

All patients were submitted to a careful interview and a standard neurological examination, including thorough bedside sensory testing. The FM patients completed the fibromyalgia-linked invalidity [26] (FIQ) in accordance with previous studies [24]. A psychologist explained the questionnaire scales and modalities of the responses to all participants. The WPI (Wide Pain Index), included into the recent ACR diagnostic criteria [2] was also correlated with LEPs and SSR values. Despite the DN4 test being a reliable and easy test for the diagnosis of neuropathic pain [27], it is not included in the evaluation of FM. However, the recent hypothesis regarding the presence of peripheral neuropathy in patients affected with FM [13] could suggest the opportunity to apply this test also to cases with diffuse symptoms, in order to detect symptoms of neuropathic origin [28]. They also completed the Zung Self-Rating Depression (SDS) [29] and Anxiety (SAS) [30] scales since these are considered reliable tools for the detection of anxiety and depressive symptoms in the general nonpsychiatric patient population.

### 2.3. Sympathetic Skin Response (SSR)

The SSR was studied using the standard method [31]. The skin temperature was maintained at 32°C (room temperature stabilized at 25-26°C). A standard electromyographic active electrode was attached to the right palm and sole and the reference electrode to the dorsum of the hand and foot. The stimuli used were single electrical stimulus at the right wrist, at 0.01 A and 0.100 sec duration. This stimulation procedure was standardized in previous studies on FM and correlated syndromes [17, 19]. Stimuli were delivered unexpectedly and in random intervals between 30 and 60 sec. Five consecutive stimuli were delivered. We measure latency from the onset of the stimulus artifact to the onset of the first negative deflection and expressed in seconds. The amplitude was measured from the baseline to the maximal negative peak and expressed in mV. The response was considered absent if no consistent voltage change occurred using a sensitivity of 50 mV per division after three trials at maximum stimuli intensity. Response latencies were considered pathological when exceeding the two SD above the mean latency of the control group. The SSR habituation was considered as the percent rate of the maximal amplitude change between the fifth and the first response. A value below 1 pointed out habituation.

### 2.4. Laser Evoked Potentials

#### 2.4.1. Stimulation Procedure

The details of the procedure are reported in de Tommaso et al. [12]. The pain stimulus consisted of laser pulses (wavelength 10.6 lm) that were generated by a CO_2_ laser (Neurolas Electronic Engineering, Florence, Italy) delivered on the dorsum of the right hand and the right foot. Two series of thirty consecutive laser stimuli were then delivered to any stimulation site at an intensity level set one step (1.5 W) above the pain threshold at an interstimulus interval of 10 seconds. An interval of 5 min separated the single stimulation series, and the order of stimulation sites was randomized.

#### 2.4.2. Recording Procedure

For the detailed procedure, please refer to de Tommaso et al. [12]. We used a montage with scalp electrodes placed over the Fz, Cz, and Pz positions of the 10-20 International System referring to the nasion with the ground at Fpz, and over T3 derivation, referred to the Fz position. Two additional electrodes were positioned above the eyebrows for the electrooculogram (EOG) recording; the ground electrode was located at Fpz.

#### 2.4.3. LEP Analysis

An investigator who was blinded to the clinical condition analyzed the LEP recordings of one second, including 100 ms of prestimulus time, at a sampling rate of 256 Hz. All LEP recordings containing transient signals that exceeded 65 mV or oculomotor artifacts in any recording channel were excluded from the average by an automatic artifact rejection algorithm. Other artifacts were visually inspected. For each stimulation site, we evaluated the averages of at least 21 valid (artifact-free) responses. A grand average across the two series of stimulation was obtained for each site. The LEPs were identified based on their latency and distribution; three responses (N1, N2, and P2) were labeled according to the procedure of Valeriani et al. [32]. The N1 component was analyzed at T3 Fz, and the N2 and P2 components were analyzed at the vertex [4, 32]. The absolute latencies of the scalp potentials were measured at the highest peak of each response component. The amplitude of each wave was measured from the baseline; the peak-to-peak amplitude was taken into consideration for the vertex biphasic LEP component (N2-P2). For estimate of habituation, the sequence of the first series of responses recorded from hand and foot sites was divided into three blocks. We considered the averages of at least 7 artifact-free consecutive responses for each block, according to de Tommaso et al. [33]. We did not evaluate the N1 habituation, given that this wave is small in amplitude and would request more repetitions for reliable averaged responses. The habituation index was the percent rate of N2-P2 amplitude change between the third and the first groups of consecutive responses (3th/1th). A value below 1 pointed out habituation.

Nerve conduction studies were performed according to standard methods [34] and the procedure is detailed in our previous study [12].

In order to avoid a time-consuming procedure, we recorded both LEPs and SSR only from the right side.

### 2.5. Statistical Analysis

The LEPs and SSR features, including habituation, were compared between groups by the Student's *t*-test (Welch's *t* test) for unpaired data and unequal variances, after the assessing of normal distribution of data by the Kolmogorov-Smirnov test and unequal variances by the Levene's test. Considering the age-related changes of LEPs [35], all the statistical comparisons were corrected for the age. The Person test was employed to evaluate the correlations among the neurophysiological variables and to detect the relationships with the clinical features. The number of subjects with absent LEPs and SSR was computed in normal and FM groups, and the difference between groups was evaluated by the chi-square test. Clinical features of patients with absent LEPs and/or SSR were compared with those of the remaining patients by the Student's *t*-test for unpaired data and equal variances.

## 3. Results

The demographic and clinical characteristics of FM patients are reported in Table 1. The FM patients showed higher scores of anxiety and depression, when compared to healthy controls (Table 1). No patient presented with neurological abnormalities, including distal sensory deficit. In FM patients, both motor and sensory nerve conduction velocities and action potential amplitudes were within normal limits.

### 3.1. Laser Evoked Potentials

Seven patients among FM groups showed no detectable LEP response for at least one site of stimulation (5 for both hand and foot, 2 only for foot). All normal subjects showed clear LEPs responses for hand and foot stimulation (chi-square 4.6, *p* = 0.043).

#### 3.1.1. Latencies

**Figure 1:**
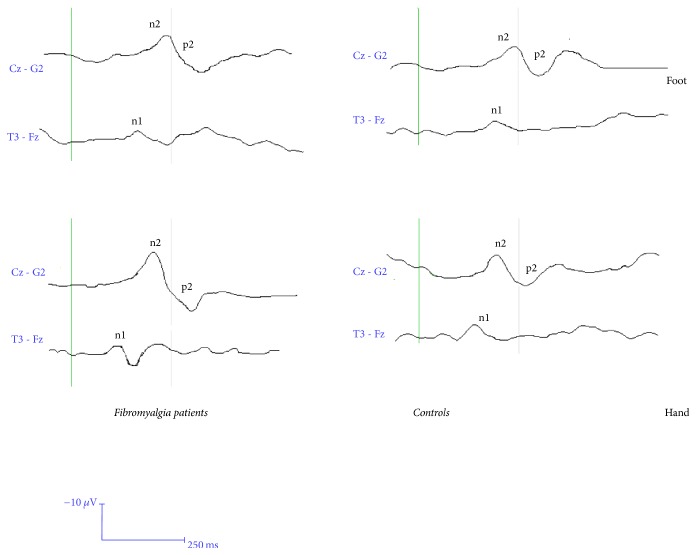
Grand average of laser evoked potentials computed in the fibromyalgia group (50) and control subjects (30). For each case the average across two consecutive series of 30 stimuli was used to compute the group LEPs.

The N1 and N2 latencies of FM patients were similar to those of controls for both stimulation sites (Table 2). The P2 latency from foot and hand was increased in FM patients compared to controls (Table 2) (Figure 1).

#### 3.1.2. Amplitudes

In FM patients the N1 and the N2 amplitude were similar to those of controls, while the P2 from both the hand and the foot was significantly increased, as well as the N2P2 vertex complex (Table 3) (Figure 1).

### 3.2. Laser Pain Rating

The subjective pain from laser stimulation of the foot was significantly increased in FM patients with respect to controls, while the VAS values from the hand stimulation were similar in the 2 groups (Table 3).

### 3.3. N2P2 Habituation

The habituation index was significantly different between patients and controls at both the hand and foot sites (Table 3). In FM patients, the mean values were above 1, indicating reduced habituation.

### 3.4. Sympathetic Skin Response

Only 1 control subject had absent SSR from at least one recording site, while in FM group 9 patients did not show clear SSR response. The chi-square test approached the statistical significance (Chi-square 3.68 *p* = 0.05).

#### 3.4.1. Latencies

**Figure 2:**
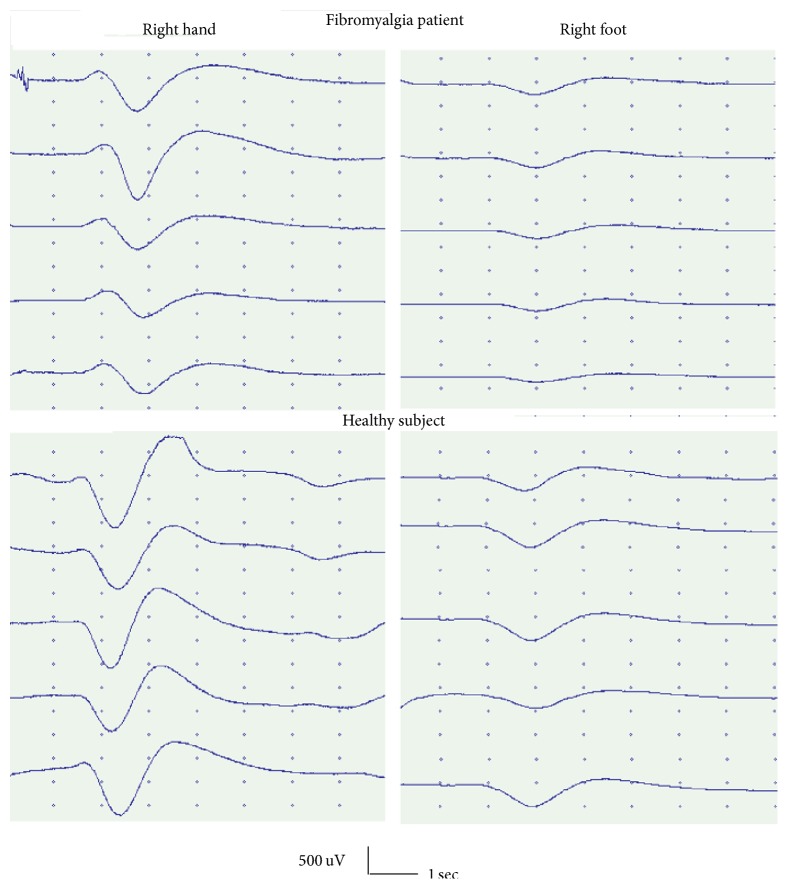
Sympathetic skin response by right median nerve stimulation in one representative fibromyalgia patient, female, 24 years old, and one healthy subject of the same sex and age.

**Figure 3:**
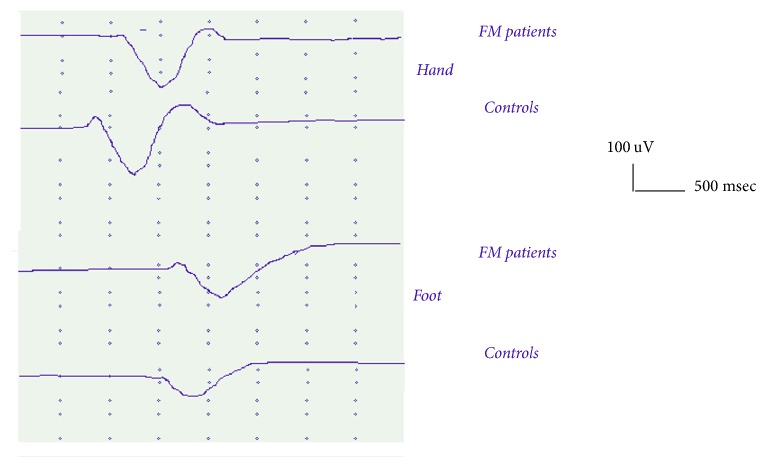
Groups' grand average of SSR from hand and foot in Fibromyalgia patients (50) and controls (30). For each case the average of the 5 responses was included to compute the groups' SSRs.

The SSR latencies from the hand and foot recording sites were significantly prolonged in FM patients (Table 4) (Figures 2 and 3).

#### 3.4.2. Amplitudes

The SSR amplitudes were similar between patients and controls, for the hand and foot stimulation (Table 4) (Figures 2 and 3).

#### 3.4.3. SSR Habituation Index

The SSR amplitude did not show a relevant reduction after repetitive stimulation, either in controls or in FM patients, who showed a slight and not significant reduction of habituation compared to healthy subjects (Table 4).

### 3.5. Correlation among Neurophysiological Variables

**Figure 4:**
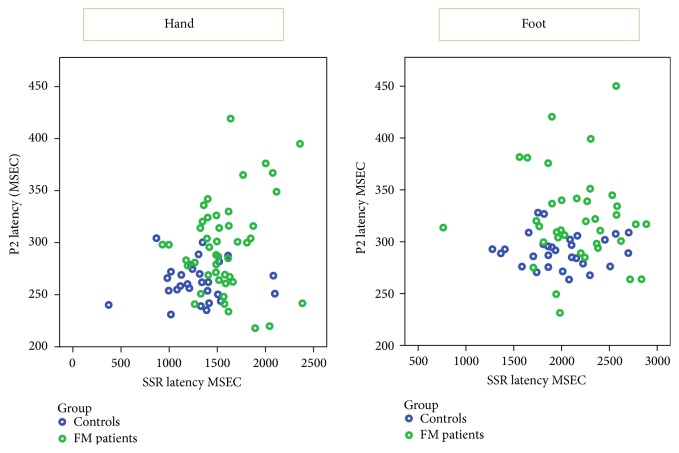
Linear dispersion plots between laser evoked potentials P2 and sympathetic skin response (SSR) latencies in fibromyalgia (FM) patients and controls.

The P2 latency and amplitudes were positively correlated in patients and controls (Pearson correlation: controls 0.535 *p* = 0.003 for hand, 0.399 *p* = 0.015 for foot; FM patients 0.387 *p* = 0.011 for hand, 0.377 *p* = 0.014 for foot), as well as the SSR and P2 latencies (Pearson correlation: controls 0.377 *p* = 0.018 for hand, 0.388 *p* = 0.016 for foot; FM patients 0.366 *p* = 0.022 for hand, 0.371 *p* = 0.015 for foot) (Figure 4).

### 3.6. Correlation between Neurophysiological and Clinical Features

The WPI was higher in patients with reduced hand-N2P2 habituation (Pearson correlation: 0.29 *p* = 0.029). The SSR latency was correlated with anxiety scores (Pearson correlation: SSR hand 0.33 *p* = 0.012; SSR foot 0.012 *p* = 0.039), while LEP latencies and amplitudes were not correlated with clinical features.

### 3.7. Clinical Characteristics of Patients with Absent LEPs/SSR

Sixteen patients did not present measurable SSR or LEPs at least on one recording site. This number was significantly higher in respect to controls (chi-square 9.2 *p* = 0.002). However, in no patient were LEPs and SSR both absent. The patients with absent LEPs or SSR showed more severe disability and higher DN4 score (Table 5). Also, in this subgroup of patients, both motor and sensory nerve conduction velocities and action potential amplitudes were within normal limits.

## 4. Discussion

This study confirms the presence of SSR anomalies in a group of patients with fibromyalgia. The most evident abnormality in FM patients was the increase of SSR latency, also correlated with the increased latency of the LEP P2 component. This would suggest a common mechanism underlying these neurophysiological patterns. Furthermore, in a subgroup of patients, specifically in the 32%, the absence of at least one of the considered responses, LEPs or SSR, was observed, associated with severe disability. The reported results confirm that FM is a complex disease characterized by phenotypic heterogeneity in the functional involvement of nociceptive and vegetative systems at peripheral and central level. The following sections deal with the detailed discussion of main data.

### 4.1. LEPs and SSR Amplitude and Latency

The most consistent LEP finding was the abnormality of the P2 component, whose amplitude increase would condition the increase of the vertex N2P2 complex. These anomalies, observed at upper and lower limbs recording sites, seem to underlie a prevalence of a central dysfunction of laser stimuli processing, differently from recent results [12]. The majority of studies pointed out an increase rather than a reduction of amplitude of the LEPs, in particular of late components N2 and P2 [7, 10]. The P2 component, which appeared increased in amplitude, would seem to originate from the anterior cingulate cortex [36, 37] responsible for the mechanisms of attention and emotional-affective significance of pain, which is hyperactive in patients with FM [38]. The P2 latency increase is somewhat unexpected, but confirmatory of the phenotypic complexity of patients with FM. Given the close correlation between increased latency and amplitude of the P2 component for all stimulation locations, already observed in normal samples [35], even this finding is attributable to a larger cortical activation and therefore a slower and cumbersome cortical elaboration under painful stimulation. The increase of P2 latency and amplitude observed in the FM group is in evident contradiction with our previous results [12], confirming that the LEP variability is characteristic of the disease. In fact, the statistic comparison of the P2 wave amplitude was barely in the range of significance, as the FM group included also patients with absent LEPs. The different exclusion criteria here used in respect to previous studies, certainly influenced the results. The exclusion of patients with any previous history of rheumatic and immunological diseases would reduce the possibility of a peripheral sensory nerves sufferance and cause the prevalence of those neurophysiological features attributable to a central dysfunction of pain processing [15]. The presence of cases with peripheral nerves involvement and pain of neuropathic origin among FM groups is frequently reported in the clinical assessment of such patients [13]. The present results seem to suggest that in the balance between the dysfunction of pain processing at the central level, and the reduced input from the periphery due to small fibers sufferance, the first abnormality prevails in determining the LEP pattern, at least in our selected FM patients. Patients with prevalent peripheral sufferance presented with complete absence of LEPs or SSR, as discussed below. A tendency for an increased laser pain perception in FM group, which was significant at the lower limb, confirmed the diffuse hyperalgesia characterizing this complex syndrome [7–10], though the lack of correlation with LEP amplitude further defined that different mechanisms underlay subjective pain and pain-related cortical responses [4, 35]. Coming to the main topic of our study, that is, the SSR, this study confirms what is generally described in patients with FM, an increased latency of sympathetic skin response in all the recording sites. The specificity of this finding for the diagnosis of FM was confirmed with complex statistical methods [19], which suggested the presence of a sympathetic system vegetative dysfunction. This dysfunction was often attributed to the FM psychopathological traits, particularly anxiety and depression [39], and found in other pathological conditions often associated with FM [40]. The results of this study confirm the correlation between SSR latency and anxiety, and therefore the source of this anomaly in a vegetative dysfunction at the central level. This would result in a delayed response to electrical stimulation, as a sign of substantial behavioral inadequacies caused by the psychopathological condition. The correlation between sympathetic skin response and LEP P2 latency suggests that similar attentional mechanisms and cortical alert may underlie both the response to pain and the vegetative reactions characteristics of the clinical picture of fibromyalgia.

The sympathetic system oversees the vegetative reaction linked to mental and emotional stress, and its activation seems regulated by various cortical areas, such as the hypothalamus, the insula and the posterior and anterior cingulate cortex [41], the latter recognized as one generator of the P2 LEP component [36]. Neuroimaging studies have confirmed the existence of a common cortical network underlying the vegetative emotional reaction and response to pain [42, 43], which may partly explain the observed correlation between the late response to the laser stimuli and the sympathetic skin response.

LEPs and SSR habituation are in accord with previous studies [10]; the present results confirmed a deficit of LEP N2P2 habituation in FM patients, while the SSR response did not show a tendency to habituate during repetitive stimulation either in patients or in controls. Habituation of the response to a repeatable stimulus, even when applied at random intervals, is a well known feature of the SSR, but the degree of habituation varies between individuals [44–47]. The lack of habituation across consecutive SSRs from electrical stimuli was described in normal subjects submitted to a standard protocol of stimulation [48]. Other studies indicate that, while the process of long-term habituation to painful heat stimuli is a common feature in normal subjects, the sympathetic nervous system shows variability in the phenomena of short and long-term habituation [49]. Habituation phenomenon is especially evident in normal subjects exhibiting biphasic responses and most frequently consists of a change of wave morphology more than reduction in the amplitude of the first negative component of the potential [47]. Here we can confirm the lack of the first negative component of SSR changes across consecutive stimulations in both FM patients and controls, as the vegetative response is probably regulated by complex and variable phenomena of progressive adaptation, which determines high variability of the pattern of progressive reduction of SSR amplitude. In any case, the lack of habituation to laser painful stimuli was correlated with the diffusion of pain symptoms in the body, confirming that this neurophysiological pattern may be the counterpart of central sensitization phenomena subtending FM and associated symptoms [50].

### 4.2. Patients with Absent LEPs and SSR

Though in the present study we did not include patients with possible causes of sensory nerves involvement, the number of subjects with absence of at least one among LEP or SSR responses was significantly higher in FM groups with respect to control one. More severe FM disability and higher DN4 scores also characterized these patients. The question about the role of peripheral nerve involvement represents a hot topic in FM research [13]. Despite the SSR pattern having a low sensitivity in the screening of small fiber neuropathy, as compared to LEPs in association with other tests of autonomic functions [20, 21], its total absence may indicate a sort of involvement of peripheral vegetative fibers in FM pathophysiology. The clinical picture of these patients was thus complicated by some features typical of neuropathic pain, as showed by the DN4 test. After the exclusion of any secondary causes of peripheral sensory nerves sufferance, as for patients with previous immunologic diseases [15], we can suppose that a primary slight sensory and vegetative neuropathy may be part of the clinical heterogeneity of FM [51]. In this scenario the employment of questionnaires for neuropathic pain [27] in large samples of patients could probably clarify the contribution of peripheral sensory nerves involvement in the complex clinical picture of FM.

## 5. Study Limits and Uncertainties

The number of subjects recruited, the type of analysis, the different exclusion criteria, and the complexity and heterogeneity of FM phenotypes would cause some contradictory results with our recent studies. In a recent study of our group [12], the finding of reduced N2P2 amplitude was probably caused by the inclusion of subjects with previous, though not active, rheumatologic diseases, which may themselves subtend subclinical neuropathies [52]. The lack of skin biopsies, at least in patients with absent LEPs or SSR, cannot confirm the presence of a slight small fiber neuropathy in such cases, though our convincement is that the employment of this procedure in all patients would be expensive and time-consuming. Clinical assessment completed by neurophysiological methods could shed light to the complexity of this syndrome and depict the peripheral and central nervous system involvement modality in single patients.

## 6. Conclusions

An increased SSR latency was confirmed in FM patients, correlated with the LEP late wave features, as a sign of abnormal central elaboration of pain. Reduced habituation of LEPs and increased latency of SSR may be confirmed to be robust neurophysiological patterns across different FM groups, beyond differences in sample size and inclusion criteria, as signs of central dysfunction of pain and vegetative reaction processing. Absent LEPs and SSR may also underlie a small fibers involvement with clinical appearance of neuropathic features, as assessed by DN4 test. As far as evidence is increasing in respect to central and peripheral nervous involvement in FM [53], the present results could point out the opportunity to submit such patients to the neurophysiological assessment of nociceptive and vegetative systems, possibly completed by skin biopsy.

The association of both neurophysiological methods with clinical evaluation could give an aid in clarifying the peripheral and central nervous system anomalies in single patients and their possible causes, in view of a targeted therapeutic approach.

## Figures and Tables

**Table 1:** Demographic and clinical characteristics of fibromyalgia patients (FM) and controls. The results of ANOVA and chi-square tests are reported. FIQ: Fibromyalgia Impact Questionnaire; WPI: Wide Pain Index.

Diagnosis	Age	Sex	Illness duration	FIQ	WPI	DN4	SAS	SDS
FM								
M	47.320	46 F 4 M	7.2	48.89	12.86	4.2	41.4	41.3
SD	13.1513		6.21	18.65	4	1.89	7.7	8.8
Controls								
M	47.433	26 F 4 M					30.2	29.9
SD	14.9659						5.3	7.1

	ANOVA: F 1.25 n.s.	Chi-square 1.98 n.s.					ANOVA: F 11.68 *p* = 0.001	F: 11.9 *p* = 0.0009

**Table 2:** Mean and standard deviations of LEP latencies in control subjects and fibromyalgia (FM) patients. The LEPs from the hand and the foot were absent in 5 patients, while 2 patients did not show clear LEPs from the only foot site. The results of Student's *t*-test for unpaired data are reported.

Diagnosis	N1 hand	N2 hand	P2 hand	N1 foot	N2 foot	p2 foot
Controls						
Mean	172.83	224.43	263.2	186.63	258.26	292.13
SD	9.84	9.07	18.74	8.79	11.93	15.96
N°	30	30	30	30	30	30
Fibromyalgia						
Mean	177.88	227.61	297.4	192.9	258.66	325.73
SD	27.44	28.70	44.32	34.55	31.93	43.42
N°	45	45	45	43	43	43
Student's *t*-test						
*t*	−1.12	−0.7	−4.68	−1.05	−0.76	−4.73
*p*	n.s.	n.s.	<0.0001	n.s.	n.s.	<0.0001

**Table 3:** Mean and standard deviations of LEP amplitudes, pain threshold-Pth (expressed in Watt), pain perception-VAS (expressed in 0–100 VAS), and N2P2 habituation (expressed as habituation index) in control subjects and fibromyalgia (FM) patients. The results of Student's *t*-test for unpaired data are reported.

Diagnosis	Hand N1 uV	Hand N2 uV	Hand P2 uV	Hand N2P2 uV	Foot N1 uV	Foot N2 uV	Foot P2 uV	Foot N2P2 uV	Hand VAS	Hand Pth (Watt)	Foot VAS	Foot Pth (Watt)	N2P2 habituation- hand (%)	N2P2 habituation-foot (%)
Controls (30)														
Mean	4.16	5.33	5.77	10.10	2.86	3.34	4.87	6.41	48.50	8.4	51.57	7.6	62	56
SD	2.19	4.55	4.55	8.53	1.96	2.58	3.84	5.22	24.34	3.1	26.93	2.9	35	37
FM (50)														
Mean	4.77	6.31	8.23	14.65	3.09	4.00	6.43	10.43	54.62	8.2	64.06	7.7	103	105
SD	2.98	5.41	5.97	10.20	2.72	3.51	4.65	7.16	20.53	2.9	23.10	2.5	91	118
Student's *t*-test														
*t*	1.05	0.8	2.07	2.05	0.44	0.88	2.12	2.88	1.2	0.89	2.19	0.91	2.77	2.51
*p*	n.s.	n.s.	0.041	0.044	n.s.	n.s.	0.039	0.005	n.s.	n.s.	0.031	n.s.	0.007	0.015

**Table 4:** Mean and standard deviations of sympathetic skin response latencies (ms), amplitudes (*µ*V), and habituation index (%) in control subjects and fibromyalgia (FM) patients. The results of Student's *t*-test for unpaired data are reported.

Diagnosis	Hand ms	Foot ms	Hand *µ*V	Foot *µ*V	Hand habituation %	Foot habituation %
Controls						
Mean	1294.64	1989.05	263.84	98.45	100	86
SD	333.60	373.69	214.41	70.80	46	44
Number	29.00	29.00	30.00	30.00	29.00	29.00
FM						
Mean	1575.32	2187.45	278.65	94.66	110	89
SD	311.84	423.58	180.47	81.88	43	36
N°	49.00	42.00	50.00	50.00	49.00	42.00
Student's *t*-test						
*T*	3.74	2.034	0.33	0.21	0.94	0.86
*p*	<0.0001	0.046	n.s.	n.s.	n.s.	n.s.

**Table 5:** Mean and standard deviations of clinical variables in fibromyalgia (FM) patients with absent (A) or present (P) SSR and/or LEPs responses. FIQ: Fibromyalgia Impact Questionnaire; WPI: Wide Pain Index. The results of Student's *t*-test for unpaired data are reported.

SSR/LEPS	AG (years)	FIQ	WPI	DN4	AGE OF FM (years)
A					
Mean	50.18	56.81	12	5.1	8
SD	15.68	7.74	4.76	2.23	6.26
P					
Mean	45.97	45.66	13.12	4.1	6.82
SD	11.79	20.84	3.78	1.78	6.22
Student's *t*-test					
*T*	0.65	2.4	0.77	2.3	0.45
*p*	n.s.	0.022	n.s.	0.01	n.s.
